# Developing educational competencies for dissemination and implementation research training programs: an exploratory analysis using card sorts

**DOI:** 10.1186/s13012-015-0304-3

**Published:** 2015-08-12

**Authors:** Margaret Padek, Graham Colditz, Maureen Dobbins, Nikolas Koscielniak, Enola K. Proctor, Anne E. Sales, Ross C. Brownson

**Affiliations:** Prevention Research Center in St. Louis, Washington University in St. Louis, One Brookings Dr., St. Louis, MO 63130 USA; Division of Public Health Sciences and Alvin J. Siteman Cancer Center, School of Medicine, Washington University in St. Louis, 660 South Euclid Ave., St. Louis, MO 63110 USA; School of Nursing, National Collaborating Centre for Methods and Tools, McMaster University, 175 Longwood Road South, Suite 210a, Hamilton, ON L8P 0A1 Canada; George Warren Brown School of Social Work, Washington University in St. Louis, One Brookings Dr., St. Louis, MO 63130 USA; Center for Clinical Management Research, VA Ann Arbor Healthcare System, Ann Arbor, MI USA; Department of Learning Health Sciences, School of Medicine, University of Michigan, Ann Arbor, MI USA

## Abstract

**Background:**

With demand increasing for dissemination and implementation (D&I) training programs in the USA and other countries, more structured, competency-based, and tested curricula are needed to guide training programs. There are many benefits to the use of competencies in practice-based education such as the establishment of rigorous standards as well as providing an additional metrics for development and growth. As the first aim of a D&I training grant, an exploratory study was conducted to establish a new set of D&I competencies to guide training in D&I research.

**Methods:**

Based upon existing D&I training literature, the leadership team compiled an initial list of competencies. The research team then engaged 16 additional colleagues in the area of D&I science to provide suggestions to the initial list. The competency list was then additionally narrowed to 43 unique competencies following feedback elicited from these D&I researchers. Three hundred additional D&I researchers were then invited via email to complete a card sort in which the list of competencies were sorted into three categories of experience levels. Participants had previous first-hand experience with D&I or knowledge translation training programs in the past. Participants reported their self-identified D&I expertise level as well as the country in which their home institution is located. A mean score was calculated for each competency based on their experience level categorization. From these mean scores, beginner-, intermediate-, and advanced-level tertiles were created for the competencies.

**Results:**

The card sort request achieved a 41 % response rate (*n* = 124). The list of 43 competencies was organized into four broad domains and sorted based on their experience level score. Eleven competencies were classified into the “Beginner” category, 27 into “Intermediate,” and 5 into “Advanced.”

**Conclusions:**

Education and training developers can use this competency list to formalize future trainings in D&I research, create more evidence-informed curricula, and enable overall capacity building and accompanying metrics in the field of D&I training and research.

**Electronic supplementary material:**

The online version of this article (doi:10.1186/s13012-015-0304-3) contains supplementary material, which is available to authorized users.

## Background

The field of dissemination and implementation (D&I) research has grown significantly over the past 15 years, as illustrated by the proliferation of frameworks and models [1], an increasing number of empirical studies [2], and dedicated federal funding [3]. Despite this growth, there remains limited capacity for training in D&I research [4, 5]. Few universities offer structured training programs in the field of D&I research science, and post-doctoral institutes often have to fill those training gaps [6, 7]. The need to increase this training capacity has been expressed at the national level within the USA through National Institutes of Health (NIH)-sponsored workgroups and meetings calling for more access to training for all levels of D&I researchers [8].

To remedy these gaps, a small number of D&I research training programs have been established and successfully launched over the past few years, including the Implementation Research Institute (IRI), the NIH Training Institute for Dissemination and Implementation Research in Health (TIDIRH), and the University of San Francisco’s Implementation Science Certificate program [4, 5, 8]. However, there is no consistent curriculum across these various programs. While their basic core elements are similar (e.g., theory, design, measurement, stakeholder engagement), a specific, crosscutting set of competencies has yet to emerge from these programs [4, 5, 8, 9]. We have set out to develop our curriculum for shorter term trainings that can appeal to a wider variety of researchers [9]. Many of those who seek additional D&I science training are individuals who already hold advanced degrees or training (MD and PhD, primarily) and need additional training in D&I research to supplement their current skill set.

Previous publications reporting their curricula provided a baseline of D&I knowledge [10, 11] and outlined how trainings should be structured. Straus et al. [11] identified the Medical Research Council Framework for Complex Interventions and the Knowledge to Action Cycle as fundamental frameworks for such training programs throughout Canada [12]. While there are over 60 models and conceptual frameworks for D&I research, there is no overarching set of educational competencies to bridge these frameworks [1], nor does the field have a consistent set of research competencies common to training programs. Competencies for D&I programs are needed urgently as continued demand for such training programs increases.

Competency-based education, which has become the norm for many research and practice-oriented training programs [13–15], has considerable appeal for developing fields such as dissemination and implementation research. Competency-based training provides clearer, potentially more rigorous, and uniform standards, as well as allows for the potential of credentialing for the future direction of these training programs [16]. The literature supporting competency-based education suggests that the utilization of competencies allows for objective parameters on which to base achievement and to gauge the growth of the researcher [17]. Training programs can use these milestones to measure their effectiveness [16, 17]. Competencies allow programs to be flexible to fit the constraints of time as well as the needs of the individual trainees [17].

Competencies can provide direction and support for overall professional development and growth. The field of D&I is still relatively new and is sometimes difficult to define [18, 19]. A set of stated competencies within a training program helps select which concepts the researchers should master in order to demonstrate proficiency [17]. This allows setting of approachable objectives for measuring progress in a D&I research career. Competencies offer structure and target points for learning achievement but can also be fluid constructs. As learning objectives are achieved, skill sets can be redefined, thus allowing for continual development and growth [17].

As part of the development of a new NIH-funded D&I research training program, entitled Mentored Training for Dissemination and Implementation Research in Cancer (MT-DIRC), we defined and refined a set of D&I competencies based on the input of established researchers in the field as well as early-stage trainees. This paper reports the findings of two phases of this competency development project. The first phase consisted of compiling competency suggestions from a panel of D&I experts throughout the world. The second phase focused on a digital card sort asking experts in the field to organize these competencies into learning levels, as they would best fit into a curriculum. We then describe the findings from that process and discuss the future direction of D&I training based on these results and related literature.

## Methods

### Phase 1: initial competency list

The MT-DIRC principal investigator (PI) and core faculty established an initial list of 33 D&I competencies for the program’s grant application developed during the fall of 2012. These competencies were based on the core faculty’s previous experience with planning and coordinating prior D&I training programs (the Implementation Research Institute (IRI) and the Training Institute for Dissemination and Implementation Research in Health (TIDIRH)) [4, 5]; recommendations from the Institute of Medicine [20]; and the faculty members’ previous endeavors with their textbook, *Dissemination and Implementation Research in Health: Translating Science to Practice* [10]. These were the primary sources that helped inform this initial list of competencies. This group consisted of seven researchers from across the USA and Canada who not only have been among national and international leaders in the fields of D&I science but also come from various disciplines such as social work, nursing, behavioral science, health communication, medicine, and epidemiology. All core members have had prior experience as instructors in D&I training programs.

To refine and amend the initial competency list, we engaged additional experts in the field of dissemination and implementation science. This additional group fits with the recommendation from the Council of the National Postsecondary Education Cooperative’s report that stakeholders in educational programs should have a role in the identification of such competencies [21]. For this particular program, our stakeholders are additional researchers who would be recipients or providers of such training. The core members each recommended names of colleagues who were also considered experts in the field of D&I science. This list consisted of 26 individuals who were from various institutions in the USA, Canada, and Australia. These additional experts all received an email from the PI with this initial list. In these emails, recipients were asked to review the attached list (initial 33 competencies) and “provide input: additions to the list, deletions, or rewording/questions about clarity.” In all, 16 different reviewers (62 %) gave unique comments or suggestions about the initial list.

Individual feedback was compiled into an Excel document and coded according to the suggested wording and action (deletion, addition, clarification). Many of the responses from the reviewer were similar in context or repetitious. The cumulative feedback from colleagues generated a list of over 100 different statements. Based on redundancy of competencies and to express clarity of meaning, the list was edited down by the PI, core faculty, and program coordinator to a final list that consisted of 43 D&I competencies. Some statements were also combined based on the similarity of concepts to provide a more feasible list that could be incorporated into a curriculum.

Based on prior experience within previous training programs, these initial competencies were categorized into four domains [4, 5]: Definition, Background, and Rationale; Theory and Approaches; Design & Analysis; and Practice-Based Considerations. A fifth domain was created which addressed Grant Development-specific competencies. While the Grant Development-specific competencies were believed to be pertinent to a training curriculum, the analysis hereafter does not focus on these competencies, as they are not content-specific to D&I research.

### Phase 2

We next categorized the newly identified 43 statements into hierarchical training levels. To do this, the team employed the use of a card sort approach. Card sort participants should be those who would be the most likely users of a particular program and allow for the categorization of knowledge within a training program [22]. In this case, we utilized previous participants in D&I training programs as they would have familiarity with the content area as well as the type of program curriculum being proposed. Because D&I science is not a general knowledge area for all health researchers, the research team engaged participants who would at least have a base knowledge of the field in order to garner more accurate results from the card sort. The use of attendance lists from previous training programs was the most complete and feasible method for obtaining a broad list of card sort participants.

### Card sort participants

Participants in the card sort were recruited through contact lists from the 2010–2013 IRI (R25MH080916) training program (*n* = 55), as well as from the 2013 TIDIRH (*n* = 65). Participants were also added from an internal Washington University D&I network listserv (*n* = 87). The research team identified the remaining participants from their previous collaborations in D&I work (*n* = 93), particularly those individuals located outside the USA. After a participant’s contact information was compiled and identified for the D&I competency card sort, potential participants were sent an email explaining the project and inviting them to participate in this activity. This activity received exempt status from Washington University’s Institutional Review Board.

### Card sorting

We used a card sort to help organize the identified D&I competencies by skill level [20]. Card sorts are widely used today by technology companies for website development and organization in user testing. However, social science researchers have been using this type of activity within participatory-based research for many years [20] as a means to organize concepts in a way that the community served by the intervention is best able to understand [21, 22]. The MT-DIRC research team took this same approach, having those who have experienced D&I training programs organize competencies into learning levels that seemed the most logical to the individual.

Qualtrics©, a web-based survey technology provider, was used to execute the virtual card sort activity. The previously identified competency list from phase 1 was used as statements within Qualtrics© in their “Question & Sort” feature. Participants were asked to place each competency statement into the column that best expressed the skill level needed to address that particular competency. Columns were marked “Beginner,” “Intermediate,” and “Advanced.” Definitions were not given for these labels to participants. The researchers intentionally chose not to define the categories to allow the participants to self-define, which gives insight into how these competencies are conceptualized into learning levels [22]. Lack of term definitions also helps to exclude any unintended bias from the research team (who are themselves considered D&I experts) on how they would define learning levels [22].

Participants were given 3 weeks to complete the card sorting. Reminder emails, a week before the close date, cued the participants to either start or finish the activity. At the end of the card sort activity, participants were asked to denote their own level of expertise in the field of D&I research (beginner, intermediate, advanced), as well as indicate their country of origin. These were the only two demographic questions asked of participants in phase 2. Participants also had the opportunity to leave comments and feedback about the list. The software tracked the amount of time it took to complete the activity with the average completion time being approximately 15 min.

### Data analysis

This activity used an exploratory analysis, employing qualitative feedback to set up the content of the activity and quantitative methods to assess the overall categorization process. While cluster and hierarchical analysis can be used during the card sort process, the group used descriptive analyses and analysis of variance (ANOVA) for this particular analysis [23, 24]. This statistical analysis was possible because of the closed nature of the card sort (i.e., the groups to which statements were sorted were predetermined) [24, 25]. After participants sorted the competency statements and data compiled, mean scores were calculated for these groupings. Competency statements placed within the beginner, intermediate, or advanced groups were coded as “1, 2, or 3,” respectively. These numbers represented categorical coding rather than ordinal coding in order to create cutoffs between the different experience level groups. The research team intended to see which competencies on average fell into which learning levels, not to determine an ordinal rank of their difficulty. The mean score was then calculated for each competency statement.

Competencies were sorted by their mean score in ascending order and then divided into tertiles based on the distribution of scores. The first tertile included competencies that on average were sorted within the beginner level. The second tertile corresponded to competencies that on average were sorted within the intermediate level. The last tertile included competencies that on average were placed within the advanced level. Once tertiles were determined, minor adjustments between groups were made based on the frequency distribution of the responses. Despite the tertile cutoffs, some distributions of competencies had a higher response rate in one category that ultimately did not result in their tertile ranking within that category. The core team examined the frequency of scores for that particular competency, and if there was a higher distribution of scores in a different experience level than what the mean score placed the competency, then adjustments were made to better reflect the majority of responses within that particular competency.

Competencies were then placed back into their original categorical domain (as explained previously). See Table 1 for a complete list of the competency groupings. One-way ANOVA was used to determine if there were statistically significant differences in how respondents grouped competencies based on their self-reported expertise level. Significant differences in responses based on country of origin could not be tested due to a small number of international participants in the activity. For the data analysis, the team used IBM SPSS Statistics Version 22.

**Table 1:** Demographics of participants in phase 2 of the card sort (*n* = 124)

What do you consider is your level of expertise in D&I/KT research?		
Level	Response	%
Beginner	36	29
Intermediate	58	47
Advanced	30	24
In what country is your home institution located?		
Country	Response	%
USA	106	86
Canada	9	7
Australia	6	5
UK	1	<1
Other	1	<1
Did not respond	1	<1

## Results

The response rate among the 300 D&I researchers contacted in phase 2 was 41 % (*n* = 124). Regarding response dynamics, among the 124 participants, 48 % completed the activity within a week of the invitation being sent out. An additional 35 % completed the survey the following week and the final 17 % by the end of the third week. The level of D&I expertise self-reported by participants fell into a relatively normal distribution: 29 % indicated a “Beginner” level, 47 % indicated an “Intermediate” level, and 24 % indicated an “Advanced” level (Table 1). Most respondents were from the USA with 14 % of participants indicating they were located outside the USA (Table 1). Two competency statements “Identify existing gaps in D&I research” and “Describe how to frame and analyze the context of D&I as a complex system with interacting parts” showed a significant difference in their level of grouping based on the respondents’ self-reported expertise level (*p* < 0.05). A Bonferroni correction was applied resulting in setting the significance level at *p* < 0.01, and these statements were no longer significant. Due to the small number of participants outside the USA (*n* = 14), the sample size was not sufficient to examine differences based on country of origin.

Most competencies (*n* = 27) fell in the “Intermediate” range. The next largest grouping was within the “Beginner” level with 11 competencies. The “Advanced” level had the smallest amount of competencies with only 5 (Table 2 and Additional file 1). Additional file 1 reports the breakdown of each competency mean score as well as the ANOVA significance between expertise levels and the competencies.

**Table 2:** Dissemination and implementation research competencies by domain

Number	Competency	Expertise^a^
Section A: Definition, Background, and Rationale		
A1	Define and communicate D&I research terminology.	B
A2	Define what is and what is not D&I research.	B
A3	Differentiate between D&I research and other related areas, such as efficacy research and effectiveness research.	B
A4	Identify the potential impact of disseminating, implementing, and sustaining effective interventions.	B
A5	Describe the range of expertise needed to conduct D&I research (e.g., mixed method experience, economic, organizational, policy, clinical).	B
A6	Determine which evidence-based interventions are worth disseminating and implementing.	I
A7	Assess, describe, and quantify (where possible) the context for effective D&I (setting characteristics, culture, capacity, and readiness).	I
A8	Identify existing gaps in D&I research.	I
A9	Identify the potential impact of scaling down (aka de-implementing) an ineffective but often used intervention.	I
A10	Formulate methods to address barriers of D&I research.	I
Section B: Theory and Approaches		
B1	Describe a range of D&I strategies, models, and frameworks.	B
B2	Identify appropriate conceptual models, frameworks, or program logic for D&I change.	I
B3	Identify core elements (effective ingredients) of effective interventions, and recognize risks of making modifications to these.	I
B4	Describe a process for designing for dissemination (planning for adoption, implementation, and sustainability during the intervention development stage).	I
B5	Describe the relationships between various organizational dimensions (e.g., climate, culture) and D&I research.	I
B6	Explain how knowledge from disciplines outside of health (e.g., business, marketing, and engineering) can help inform further transdisciplinary efforts in D&I research.	I
B7	Identify and articulate the interplay between policy and organizational processes in D&I.	I
Section C: Design & Analysis		
C1	Describe the core components of external validity and their relevance to D&I research.	B
C2	Identify common D&I measures and analytic strategies relevant for your research question(s).	B
C3	Identify and measure outcomes that matter to stakeholders, adopters, and implementers.	I
C4	Describe the application and integration of mixed-method (quantitative and qualitative) approaches in D&I research.	I
C5	Apply common D&I measures and analytic strategies relevant for your research question(s) within your model/framework.	I
C6	Identify possible methods to address external validity in study design reporting and implementation.	I
C7	List the potential roles of mediators and moderators in a D&I study.	I
C8	Identify and articulate the trade-offs between a variety of different study designs for D&I research.	I
C9	Describe how to frame and analyze the context of D&I as a complex system with interacting parts.	I
C10	Effectively integrate the concepts of sustainability/sustainment and the rationale behind them in D&I study design.	I
C11	Describe gaps in D&I measurement and critically evaluate how to fill them.	A
C12	Effectively explain and incorporate concepts of de-adoption and de-implementation into D&I study design.	A
C13	Incorporate methods of economic evaluation (e.g., implementation costs, cost-effectiveness) in D&I study design.	A
C14	Evaluate and refine innovative scale-up and spread methods (e.g., technical assistance, interactive systems, novel incentives, and “pull” strategies).	A
Section D: Practice-Based Considerations		
D1	Describe the importance of incorporating the perspectives of different stakeholder groups (e.g., patient/family, employers, payers, healthcare settings, public organizations, community, and policy makers).	B
D2	Describe the concept and measurement of fidelity.	B
D3	Articulate the strengths and weaknesses of participatory research in D&I research.	B
D4	Determine when engagement in participatory research is appropriate with D&I research.	I
D5	Describe the appropriate process for eliciting input from community-based practitioners for adapting an intervention.	I
D6	Identify and apply techniques for stakeholder analysis and engagement when implementing evidence-based practices.	I
D7	Identify a process for adapting an intervention and how the process is relevant to D&I research.	I
D8	Explain how to maintain fidelity of original interventions during the adaption process.	I
D9	Identify sites to participate in D&I studies, and negotiate or provide incentives to secure their involvement.	I
D10	Identify and develop sustainable partnerships for D&I research.	I
D11	Describe how to measure successful partnerships for D&I research.	I
D12	Use evidence to evaluate and adapt D&I strategies for specific populations, settings, contexts, resources, and/or capacities.	A

^a^Expertise levels: B = Beginner; I = Intermediate; A = Advanced

## Discussion

This study identified and assigned 43 D&I research competencies into three experience levels as well as sorted them into four topical domains. Some domains did not contain “Advanced” level competencies (Definition, Background, and Rationale and Theory and Approaches), and the distribution of expertise levels overall across the four domains was not equal. The heavier emphasis on intermediate competencies may be an indication that the field is growing and researchers are still unclear of what constitutes advanced-level D&I knowledge. However, arrangement of these competencies within levels provides training programs with guidance on how to structure content based on the progression of the content within a curriculum.

While the overall response rate of 41 % (*n* = 123) may seem low for this activity, card sorting literature suggests that saturation for card sorts begins around *n* = 30 participants [23, 26]. It is more important that the participants have characteristics that are most relevant to the use of the intended outcome of the card sort [26]. In this case, it was important to employ participants who have been users of D&I training programs in the past in order to generate robust results.

Work with card sorts have previously allowed researchers to identify the needs of particular groups for developing evidence-based trainings [27]. However, card sorts had not been utilized in the context of D&I training curriculum development. Though Straus’s adaptation of Graham and colleagues’ Knowledge to Action (KTA) Cycle provides one basis for D&I training competencies, the KTA framework does not identify specific content that should be covered in training programs [11, 12]. In future work, educators should focus on understanding how this card sort work and the KTA framework relate to each other.

Many of the participants in this study, both from the initial list gathering to the final card sort activity, have been previous attendees (trainees or faculty) of D&I training programs. While previous development of training program curricula have been an accumulation of experienced researchers’ knowledge [5, 6], our integration of attendees’ and trainees’ feedback helps provide insight into perceived training needs [18]. While the expertise of advanced-level D&I experts is critical, it is also important to gauge how these competencies are viewed by individuals at lower levels of D&I expertise. Since many of these trainees are newer to the field of D&I, their perception of the competencies may be critical to the overall success of how future training programs are framed [25]. By using ANOVA to assess differences between groups, we were also able to determine that there were no significant differences in the way that competencies were sorted based on self-reported expertise levels. As a result, we consider those ratings by individuals at lower levels of expertise just as useful as those who are considered more advanced within the field of D&I science [25].

### Implications for training programs

The University of California, San Francisco has laid a framework for a curriculum that is utilized in their concentration and certificate degree programs [9]. Due to the timing of their publication and the initiation of this project, their curriculum was not used to inform ours. However, there are many overlapping themes and concepts between the competency lists [9]. Owing to their call to expand the work they have started, these competencies have begun to address the expansion of their work. To our knowledge, this list of D&I competencies is the first that has been systematically developed by a wide audience of current researchers within the field.

Participants have identified, based on their conceptualization of the competencies, where and when these particular skills should be addressed in the progression of a curriculum. While the majority of the participants had terminal degrees, the card sort activity was presented in a way that the competencies could be adapted for any level of learner. These competencies may equally inform the development of entry-level and mid-level courses that are likely offered in public health or other training programs. By assessing the learning levels for each competency, priorities can be set on specific content depending on the target audience of the different types of training (e.g., masters-level students, doctoral-level students, post-doctoral trainees).

In MT-DIRC, we have adopted this set of competencies to guide the MT-DIRC training and we are assessing how well trainees progress according to these competencies. Upon the inaugural cohort’s entry into the program, they were given a pre-assessment asking them to rate “How skilled do you currently feel in the following D&I competencies” for each of the competencies based on a 5-point scale. The MT-DIRC fellows were given a follow-up post-assessment at 6 months after attendance at their first institute and will be given another post-assessment at 18 months post-initial institute. We plan to conduct these same assessments with subsequent cohorts. We have used the assessment to gauge how the agenda of the institute should be structured to best fit the training needs of the fellows.

While previous programs have used publications, presentations, and grant submissions as metrics of a program success [5], these do not allow fine-grained measurement of knowledge acquisition by trainees. The potential to use competencies as an assessment tool also allows for the ability to customize training programs based on the assessed needs of the trainees prior to their entry in the program (pre- and post-test assessments) and track overall performance. Competency assessments still need to be developed and validated, but our contribution provides an initial starting point for this work.

### Study limitations

The use of tertile rankings to establish the cutoff for the competencies may not be the ideal method for establishing groupings. However, because of the nature of the card sort software, as well as the inability to provide in-person direction and feedback as is common with traditional card sorts [24], tertile groupings were our best alternative. This method did not take into consideration the frequency of distribution within each competency score, hence the need to make manual adjustments based on a sometimes heavier emphasis of a competency in one expertise level over another. Since the card sort activity was done virtually rather than in person, the team could not gauge responses from participants as to why they placed heavier emphasis on the “Intermediate” category. Previous card sorts have shown that feedback from participants and a debrief session after the activity provide additional insight into the thought processes of participants as well as identify mediating factors [24, 27].

It is also possible that because the field of D&I research is still relatively new, the concept of “Advanced” competencies may be unclear to the experts. Literature suggests that expertise levels of participants have an effect on the way categorization of the concepts occurs [25]. Despite evidence from the previous literature, we found in our study that expertise levels did not have an overall significant effect on the way competency statements were grouped. We did find an uneven distribution of self-identifying expertise levels. “Advanced” participants were the smallest group (24 %), and the “Intermediate” participants (47 %) were the largest, suggesting a byproduct of the newness of the field or the sampling methods used for this activity.

Finally, the lack of participation by D&I researchers outside the USA poses a limitation. While our team attempted to obtain international participation in the card sort, only 14 % of respondents indicated they were from countries outside of the USA. With many countries actively pursuing the field of D&I, it would have been useful to have a larger response from non-US respondents as they could provide insight into the field of D&I training from other cultural and social perspectives. The fields of dissemination and implementation science are emerging along somewhat parallel paths in different countries, and a unified set of competencies and recommendations for training would ideally include expert guidance from across the globe. It is important to note that most D&I research is occurring in higher income countries and there is a need to engage low- and middle-income countries to provide a more globally relevant view of competencies.

### Next steps

The current results provide a foundation for future training programs; however, further rigorous testing is needed for these competencies. Competency sets are not a rigid structure but rather a fluid compass [17]. As new technologies and methodologies are developed, training competencies should be reexamined periodically as well to parallel the ever-evolving social and research climate. An additional element of stakeholder and practitioner definitions can also be added to the development and depth of these competencies, since this study only looked at D&I competencies from the perspective of researchers. Since the aim of D&I science is to better translate research into real-world practice, it is vital that we also explore the training of researchers from the perspective of a broad array of stakeholders [6].

These competencies need to be tested on a more global scale. With the long establishment of Knowledge Translation (KT) Canada’s Summer Institute, and with a strong emphasis on KT and implementation science in countries such as the UK, the Netherlands, and Australia, it is imperative to analyze how these competencies measure up with their current perspectives of D&I skills [11]. As the research climate has become more transnational, standards within training programs should aim to reach a wider audience. This transnational audience needs to include low- and middle-income countries as well to ensure that competencies are relevant to a broader audience. This need appears to be growing. One of the largest US-based D&I training programs, TIDIRH, has seen their number of low- and middle-income country applicants double in the years since its inception (5 middle-low-income international applicants in 2011 to 13 middle-low-income international applicants during their 2014 application cycle).

## Conclusion

Our findings provide a basis for a more structured curriculum for D&I training programs. These competencies provide the ability to critically assess current programs and provide the needed structure for future D&I training programs, while also allowing flexibility for continual growth and development that is likely to occur within the field. These competencies also help to identify the needs of trainees to facilitate their professional growth and to provide the ability to tailor training to fit the specific needs of the trainees. Most areas within the field of social sciences are moving towards competency-based education, and the fields of D&I science should be no different. These competencies are foundational tools that will be necessary to further build capacity in the field of D&I research and strengthen the training of the next generation of D&I researchers.

## Additional file

Additional file 1: Table S1. Dissemination and implementation research competencies by skill level. This table contains a more detailed breakdown of the data for each competency.

## Abbreviations

D&I: dissemination and implementation
IRI: Implementation Research Institute
KT: Knowledge Translation
KTA: Knowledge to Action
MT-DIRC: Mentored Training for Dissemination and Implementation Research in Cancer
PI: principal investigator
TIDIRH: Training Institute for Dissemination and Implementation Research in Health
