# Supplementary material for: Developing educational competencies for dissemination and implementation research training programs: an exploratory analysis using card sorts
Source: Implement Sci. 2015 Aug 12;10:114. doi: 10.1186/s13012-015-0304-3 (PMC4534127; doi:10.1186/s13012-015-0304-3)
Supplement: Additional file 1: Table S1. — Dissemination and implementation research competencies by skill level. This table contains a more detailed breakdown of the data for each competency. [file 13012_2015_304_MOESM1_ESM.docx]

**Additional file 1**

**Table S1 Dissemination and implementation research competencies by skill level**

| **Competency** | **Beginner Expertise Mean** | **Intermediate Expertise Mean** | **Advanced Expertise Mean** | **Overall Mean** | **Std Error** | **ANOVA Sig** |
| --- | --- | --- | --- | --- | --- | --- |
| Define and communicate D&I research terminology | 1.08 | 1.19 | 1.07 | 1.13 | 0.034 | 0.251 |
| Define what is and what is not D&I research | 1.11 | 1.19 | 1.1 | 1.15 | 0.036 | 0.505 |
| Describe the importance of incorporating the perspectives of different stakeholder groups (e.g., patient/family; employers, payers, healthcare settings, public organizations, community and policy makers) | 1.19 | 1.21 | 1.27 | 1.22 | 0.042 | 0.803 |
| Differentiate between D&I research and other related areas, such as efficacy research and effectiveness research | 1.19 | 1.29 | 1.17 | 1.23 | 0.043 | 0.426 |
| Describe the concept and measurement of fidelity | 1.33 | 1.24 | 1.13 | 1.24 | 0.049 | 0.337 |
| Describe the core components of external validity and their relevance to D&I research | 1.39 | 1.33 | 1.23 | 1.32 | 0.053 | 0.57 |
| Describe a range of D&I strategies, models and frameworks | 1.47 | 1.43 | 1.37 | 1.43 | 0.056 | 0.794 |
| Articulate the strengths and weaknesses of participatory research in D&I research | 1.47 | 1.55 | 1.63 | 1.55 | 0.055 | 0.575 |
| Describe the range of expertise needed to conduct D&I research (e.g., mixed method experience, economic, organizational, policy, clinical) | 1.5 | 1.53 | 1.67 | 1.56 | 0.063 | 0.601 |
| Identify common D&I measures & analytic strategies relevant for your research question(s) | 1.53 | 1.55 | 1.6 | 1.56 | 0.052 | 0.877 |
| Identify the potential impact of disseminating, implementing and sustaining effective interventions | 1.42 | 1.69 | 1.67 | 1.6 | 0.066 | 0.185 |
| Identify and measure outcomes that matter to stakeholders, adopters and implementers | 1.58 | 1.69 | 1.63 | 1.65 | 0.057 | 0.734 |
| Identify appropriate conceptual models, frameworks, or program logic for D&I change | 1.81 | 1.67 | 1.47 | 1.66 | 0.059 | 0.114 |
| Determine when engagement in participatory research is appropriate with D&I research | 1.61 | 1.71 | 1.8 | 1.7 | 0.056 | 0.475 |
| Identify core elements (effective ingredients) of effective interventions and recognize risks of making modifications to these | 1.72 | 1.72 | 1.73 | 1.73 | 0.058 | 0.997 |
| Describe a process for designing for dissemination (planning for adoption, implementation and sustainability during the intervention development stage) | 1.64 | 1.83 | 1.87 | 1.78 | 0.062 | 0.33 |
| Describe the appropriate process for eliciting input from community-based practitioners for adapting an intervention | 1.78 | 1.86 | 1.8 | 1.82 | 0.055 | 0.791 |
| Describe the application and integration of mixed-methods (quantitative and qualitative) approaches in D&I research | 1.86 | 1.83 | 1.83 | 1.84 | 0.06 | 0.972 |
| Apply common D&I measures & analytic strategies relevant for your research question(s) within your model/framework | 1.86 | 1.84 | 1.9 | 1.86 | 0.054 | 0.921 |
| Determine which evidence-based interventions are worth disseminating and implementing | 2.03 | 1.83 | 1.83 | 1.89 | 0.062 | 0.35 |
| Describe the relationships between various organizational dimensions (e.g., climate, culture) and D&I research | 1.89 | 1.86 | 2 | 1.9 | 0.061 | 0.662 |
| Identify possible methods to address external validity in study design reporting and implementation | 1.89 | 1.9 | 2.03 | 1.93 | 0.057 | 0.584 |
| Identify a process for adapting an intervention and how the process is relevant to D&I research | 1.83 | 1.91 | 2.1 | 1.94 | 0.059 | 0.249 |
| Assess, describe, and quantify (where possible) the context for effective D&I (setting characteristics, culture, capacity & readiness) | 2 | 1.97 | 1.9 | 1.96 | 0.061 | 0.837 |
| Identify existing gaps in D&I research | 2.08 | 1.83 | 2.13 | 1.98 | 0.055 | 0.04 |
| List the potential roles of mediators and moderators in a D&I study | 1.83 | 2.07 | 1.97 | 1.98 | 0.061 | 0.265 |
| Explain how to maintain fidelity of original interventions during the adaption process | 2.11 | 2.03 | 1.8 | 2 | 0.055 | 0.101 |
| Explain how knowledge from disciplines outside of health (e.g., business, marketing, and engineering) can help inform further trans-disciplinary efforts in D&I research | 1.86 | 1.95 | 2.3 | 2.01 | 0.072 | 0.062 |
| Identify sites to participate in D&I studies and negotiate or provide incentives to secure their involvement | 2.06 | 2.12 | 1.9 | 2.05 | 0.066 | 0.408 |
| Identify and develop sustainable partnerships for D&I research | 2.11 | 2.17 | 2.07 | 2.13 | 0.064 | 0.793 |
| Identify and articulate the trade-offs between a variety of different study designs for D&I research | 2.08 | 2.17 | 2.17 | 2.15 | 0.059 | 0.802 |
| Describe how to frame and analyze the context of D&I as a complex system with interacting parts | 1.94 | 2.17 | 2.43 | 2.17 | 0.066 | 0.027 |
| Identify and apply techniques for stakeholder analysis and engagement when implementing evidence-based practices | 2.22 | 2.21 | 2.23 | 2.22 | 0.057 | 0.982 |
| Identify the potential impact of scaling down (aka de-implementing) an ineffective but often used intervention | 2.17 | 2.24 | 2.33 | 2.24 | 0.064 | 0.644 |
| Describe how to measure successful partnerships for D&I research | 2.25 | 2.14 | 2.43 | 2.24 | 0.058 | 0.123 |
| Formulate methods to address barriers of D&I research | 2.25 | 2.31 | 2.23 | 2.27 | 0.059 | 0.844 |
| Identify and articulate the interplay between policy and organizational processes in D&I | 2.11 | 2.28 | 2.43 | 2.27 | 0.061 | 0.154 |
| Effectively integrate the concepts of sustainability/sustainment and the rationale behind them in D&I study design | 2.28 | 2.22 | 2.33 | 2.27 | 0.059 | 0.754 |
| Use evidence to evaluate and adapt D&I strategies for specific populations, settings, contexts, resources and/or capacities | 2.39 | 2.36 | 2.43 | 2.39 | 0.06 | 0.896 |
| Effectively explain and incorporate concepts of de-adoption and de-implementation into D&I study design | 2.53 | 2.47 | 2.47 | 2.48 | 0.058 | 0.89 |
| Describe gaps in D&I measurement and critically evaluate how to fill them | 2.56 | 2.41 | 2.6 | 2.5 | 0.054 | 0.319 |
| Incorporate methods of economic evaluation (e.g., implementation costs, cost-effectiveness) in D&I study design | 2.53 | 2.71 | 2.57 | 2.62 | 0.051 | 0.274 |
| Evaluate and refine innovative scale-up and spread methods (e.g., technical assistance, interactive systems, novel incentives and ‘pull’ strategies) | 2.69 | 2.67 | 2.73 | 2.69 | 0.051 | 0.896 |
